# Assessment of a novel method to detect clarithromycin-resistant *Helicobacter pylori* using a stool antigen test reagent

**DOI:** 10.1186/s12876-020-01549-9

**Published:** 2020-11-23

**Authors:** Toshihiko Kakiuchi, Kazutoshi Hashiguchi, Ichiro Imamura, Aiko Nakayama, Ayako Takamori, Masumi Okuda, Muneaki Matsuo

**Affiliations:** 1 Department of Pediatrics, Faculty of Medicine, Saga University, 5-1-1 Nabeshima, Saga-shi, Saga, 849-8501 Japan; 2 Division of Gastroenterology, Department of Internal Medicine, Imamura Hospital, Tosu, Japan; 3 Division of Clinical Research Center, Saga University Hospital, Saga, Japan; 4 Department of Pediatrics, Hyogo College of Medicine, Nishinomiya, Japan

**Keywords:** 23S rRNA, Clarithromycin resistance, Genetic mutation, Drug susceptibility test

## Abstract

**Background:**

The resistance rate of *Helicobacter pylori* to clarithromycin (CAM) is high among infected children in Japan. Therefore, a new method for detecting CAM-resistant *H. pylori* using a minimally invasive technique is strongly desired. We aimed to investigate the clinical usefulness of our newly developed nested polymerase chain reaction-quenching probe (Nested PCR-QP) method using stool specimens.

**Methods:**

We first evaluated our method using a residual solution of the *H. pylori* stool antigen test for adolescents. Then, we evaluated our method using culture testing for adults.

**Results:**

Among 57 middle school students with *H. pylori*, the Nested PCR-QP test results of 53 (90.3%) were able to be analyzed. A total of 28 students had CAM resistance mutations. We found a genetic mutation in 28 students and no mutation in 23 students, and these results were consistent with those of PCR-direct sequencing. In the 23 adults who were diagnosed with *H. pylori* infection using the rapid urease test and culture testing, we were able to use Nested PCR-QP for analyzing 21 adults who tested positive in the stool *H. pylori* antigen test. The results obtained for all 21 adults were consistent with those obtained via the drug susceptibility test.

**Conclusions:**

Our novel method could be useful for non-invasively detecting CAM resistance mutations in *H. pylori*. This may help select a drug to reduce eradication failure rates against *H. pylori*.

*Trial registration* This study was registered with the University Hospital Medical Information Network Clinical Trials Registry (no. UMIN000030632, https://upload.umin.ac.jp/cgi-open-bin/ctr_e/ctr_view.cgi?recptno=R000034977) on 29 December 2017.

## Background

*Helicobacter pylori* infects the gastric mucosa, where it causes chronic inflammation, which in turn increases the risk of gastric cancer [1–4]. Eradication of *H. pylori* reduces the risk of gastric cancer [3, 5–8]. *H. pylori* eradication is also more effective at reducing the risk of gastric cancer in younger individuals than in older individuals who have suffered from chronic gastritis over a long duration [9–14].

In Japan, While the age-standardized incidence and mortality rates of gastric cancer are decreasing in men and women, gastric cancer has the highest incidence in men and the third highest incidence in women among all cancers. Furthermore, the gastric cancer mortality rate is second highest in men and third highest in women among all cancer mortalities [15]. Recently, the number of municipalities in Japan that perform *H. pylori* testing and eradication in middle school students for preventing gastric cancer has been increasing [16, 17]. In 2016, Saga Prefecture became one of the first prefectures in Japan to begin testing for *H. pylori* in all third year middle school students in the prefecture and performing eradication therapy for those who tested positive [18]. This effort is part of the “A *H. pylori* screening and treatment program to eliminate gastric cancer among junior high school students in Saga Prefecture.” While *H. pylori* tests being conducted throughout Japan differ across municipalities, many regions use the urine *H. pylori* antibody test as the primary test and then use either the urea breath test or the stool *H. pylori* antigen test as the secondary test [19–21].

Eradication therapy for *H. pylori* is performed for those who test positive in the secondary test. Because the targeted patients are children, selection of the eradication drug is extremely important from the perspectives of safety and efficacy. The conventional treatment for *H. pylori* infection is clarithromycin (CAM) administration [22, 23]. The *H. pylori* CAM resistance rate is high in young people in Japan [24, 25]. Therefore, CAM resistance must be assessed via drug sensitivity testing before selecting an effective and safe eradication drug. However, because the drug susceptibility test used on strains that are cultured from gastric biopsy tissue involves a highly invasive test sample collection procedure, it places a heavy burden on patients. This makes performing testing in children difficult, particularly in those who are asymptomatic. The major cause of CAM resistance in *H. pylori* is 23S rRNA genetic mutations (A2142C, A2142G, and A2143G) [26]. In recent years, there have been reports on a genetic test for detecting *H. pylori* CAM resistance using a non-invasive stool test. However, the sensitivity of such tests remains insufficient (60–80%), and the specificity has not been fully evaluated [27–29].

Therefore, we developed the Nested polymerase chain reaction-quenching probe (Nested PCR-QP) method. This method is used to detect 23S rRNA genetic mutations that are associated with CAM resistance with a high degree of sensitivity using the fluid remaining after the stool antigen test. This study aimed to investigate the clinical usefulness of this novel Nested PCR-QP method that uses stool specimens.

## Methods

We designed two clinical studies to examine the clinical usefulness of Nested PCR-QP using stool specimens. The first study involved a method using the residual solution of the *H. pylori* stool antigen test for adolescents because performing esophagogastroduodenoscopy (EGD) in adolescents is difficult. The second study used culture testing for adults.

### Sensitivity of Nested PCR-QP detection and correlation with eradication therapy

The subjects were middle school students in Saga Prefecture who underwent *H. pylori* testing between August and December 2017, under our program to eliminate gastric cancer. Our program targeted 8519 students in 2016, of which 7230 actually participated. The urine *H. pylori* antibody test (RAPIRAN®; Otsuka Pharmaceutical Co., Ltd., Tokyo, Japan), which was used as the primary test, gave positive results for 356 students, of whom 120 were randomly selected and asked to participate in the present study. The consent to participate in the present study was obtained from 71 middle school students (median age was 14.7 years, male/female ratio = 40/31). These 71 students underwent stool *H. pylori* antigen testing (Testmate Rapid Pylori Antigen®; Wakamoto Pharmaceutical Co., Ltd., Tokyo, Japan) as the secondary test. Among these students, the secondary test results were positive for 57 and negative for 14 individuals. Therefore, the 57 patients who tested positive in the secondary test were judged to be infected with *H. pylori*. We then performed Nested PCR-QP measurements using the leftover specimens from the stool antigen tests that the 57 students who tested positive had undergone. DNA sequencing analysis was performed for all 57 samples by carrying out Nested PCR-QP using PCR-direct sequencing. Students who tested positive in the stool antigen test underwent a 7-day treatment that comprised 20 mg vonoprazan (VPZ) (Takeda Pharmaceutical Co., Ltd., Tokyo, Japan), 750 mg amoxicillin (AMPC), and 200 mg CAM twice a day, regardless of the results regarding CAM resistance mutations. Students who failed the primary eradication therapy were treated with a regimen that replaced CAM with metronidazole (MNZ) as a secondary eradication therapy. The regimen for *H. pylori* eradication therapy followed that specified for public medical insurance in Japan. Confirmation of eradication therapy was conducted using the urea breath test (UBIT® tablet, 100 mg, and POC One®; Otsuka Electronics Co., Ltd., Hirakata, Japan).

### The culture test and drug susceptibility test

We performed another study on adults at Imamura Hospital (Tosu, Japan) because EGD is difficult to perform in asymptomatic children. The subjects were 23 adults who were diagnosed with *H. pylori* infection on the basis of the results of the rapid urease test (RUT) (Helicocheck®; Institute of Immunology, Co., Ltd., Tochigi, Japan) and culture testing performed at the hospital. These subjects had undergone EGD between February 2018 and September 2018. Culture testing and drug susceptibility testing (*H. pylori* drug sensitivity test: *H. pylori* minimal inhibitory concentration measurement) were performed by BML, Inc. (Tokyo, Japan). We used the minimal inhibitory concentration (MIC) breakpoints recommended by the Japan Society of Chemotherapy for CAM (susceptible ≤ 0.25 µg/mL) and AMPC (susceptible ≤ 0.03 µg/mL) [30]. We also used the breakpoints listed in the EUCAST Trial breakpoint table v.8.1 for MNZ (resistant > 8 µg/mL) [31]. Stool specimens were used in the stool *H. pylori* antigen test, and the remnant solution of those who tested positive was used to perform Nested PCR-QP measurements.

### Nested PCR-QP

Nested PCR-QP is a novel genetic analysis method that analyzes 23S rRNA genetic mutations (A2142C, A2142G, and A2143G) that are associated with CAM resistance in *H. pylori*. The basic components are the Nested PCR and QProbe [32]. Stool *H. pylori* antigen test remnant solution was used in Nested PCR-QP. DNA was extracted from Nested PCR-QP samples of 100 μL using a QIAamp® DNA Mini kit (QIAGEN GmbH, Hilden, Germany) to obtain 150 μL of DNA solution. This DNA solution was the first PCR template for Nested PCR-QP. In addition, for quality control, positive and negative controls were measured simultaneously for each assay. For positive control, plasmid DNA containing a region amplified by this method in the H. pylori 23S rRNA gene was used. Two types of positive controls, wild type and mutant type (A2143G), were used. For negative control, Tris–EDTA buffer was used.

The first PCR primers were Hp23S 1835F and Hp23S 2327R based on the method described by Noguchi et al. [33]. The first PCR used a 1-μL template that was created using a T100 Thermal Cycler (Bio-Rad Laboratories Inc., Hercules, CA, USA) and it was performed using Ampdirect plus BIOTAQ HS DNA Polymerase (Shimadzu Corp., Kyoto, Japan). The PCR protocol was as follows: after primer annealing for 10 min at 95 °C, 36 cycles were performed at 94 °C for 30 s, 50 °C for 60 s, and 72 °C for 60 s.

The first PCR mixture, which was diluted 20-fold in sterilized water, was used as the second PCR template. The second PCR primers were the newly designed HP F1 (5′-CCAGAGATTCAGTGAAATTGTAGTGGAGGTG-3′) and HP R3 (5′-GGCTCCATAAGAGCCAAAGCCCTTAC-3′), and the probe used was the newly designed HP QP1 (5′-CCGCGGCAAGACGGAAAGAC-BODIPY-3′). The second PCR and melting curve analysis were performed using a 5-μL template from LightCycler Nano (Roche Diagnostics K.K., Tokyo, Japan) and KOD DNA Polymerase (Toyobo Co., Ltd., Osaka, Japan). The PCR protocol was as follows: after primer annealing for 2 min at 98 °C, 65 cycles were performed at 95 °C for 5 s and 55 °C for 15 s. Melting curve analysis was performed as follows: after the second PCR, warming was performed from 40 to 80 °C at a rate of 0.09 °C/s, and measurements were obtained using fluorescent light with a wavelength of 510–528 nm. Melting curve analysis using fluorescence quenching allowed us to determine that there were no mutations when the fluorescence quenching inflection point was between 67 and 71 °C (wild-type). However, there were mutations between 59 and 63 °C (A2142G, A2143G) and between 63 and 67 °C (A2142C).

### Statistical analysis

The proportion of CAM resistance among *H. pylori* and the effectiveness of eradication therapy were compared between the Nested PCR-QP mutation group and the non-mutation group using the chi-square test. Statistical significance was set at a *P* value of < 0.05. Statistical analyses of the data were performed using JMP Pro13 (SAS Institute Inc., Cary, NC, USA).

## Results

### Assessment of CAM resistance genetic mutations using Nested PCR-QP

The results of stool *H. pylori* antigen testing and Nested PCR-QP among the 71 students who tested positive in the urine *H. pylori* test are shown in Table 1. Of the 57 students who tested positive in the stool *H. pylori* antigen test, Nested PCR-QP analysis was possible for 53 (93.0%) students. Twenty-eight (52.8%) of these 53 students had CAM resistance mutations. One student possessed the A2142C mutation and 27 students possessed the A2143G mutation. None of the students possessed the A2142G mutation. Twenty-five students did not have any CAM resistance mutations. These results were consistent with those of PCR-direct sequencing.

**Table 1:** Determination of CAM resistance mutations using Nested PCR-QP

Fecal antigen test	Nested PCR-QP	(n = 71)
Positive	Mutation	28
	Wild type	25
	Not detected	4
Negative	Not tested	14

*PCR* polymerase chain reaction, *QP* Q probe

Investigation of the eradication therapy results in 41 students for whom we were able to conduct follow-up examinations showed that CAM resistance mutations affected eradication therapy. The eradication therapy success rate was 94.4% (17/18) in the non-mutation group and 82.6% in the CAM resistance mutation group (19/23; *P* = 0.25; Table 2). Selection of eradication therapy drugs did not change according to CAM resistance mutations. All five of the subjects for secondary eradication showed successful outcomes following eradication therapy with a regimen using MNZ.

**Table 2:** Effect of CAM resistance mutations on eradication therapy

	Eradication therapy		*P* value^a^
	Success (n = 36)	Failure (n = 5)	
Nested PCR-QP			
Mutation (n = 23)	19	4	0.25
Wild type (n = 18)	17	1	

*PCR* polymerase chain reaction, *QP* Q probe

^a^Analyzed by Fisher’s exact test

### Nested PCR-QP in relation to the culture test and drug sensitivity test

At Imamura Hospital, during the designated period, 30 patients received EGD in a general practice situation. Five of these patients gave a negative RUT result, and two of the remaining did not submit a stool sample. Therefore, 23 individuals in total were included in the present study. EGD results found no evidence of gastric ulcers, duodenal ulcers, or gastric cancer, but chronic gastritis was found in all cases. The results of culture and drug susceptibility testing, as well as the stool *H. pylori* antigen test and Nested PCR-QP, in 23 adults (median age of 53 years, male/female ratio = 12/11) with *H. pylori* infection are shown in Table 3. Drug susceptibility testing showed that the CAM resistance rate was 30.4% (7/23), the AMPC resistance rate was 4.3% (1/23), and the MNZ resistance rate was 39.1% (9/23). The sensitivity of the stool *H. pylori* antigen test was 91.3% (21/23). Nested PCR-QP was not performed for two adults with a negative stool *H. pylori* antigen test result.

**Table 3:** Coincidence rate of the Nested PCR-QP and the drug susceptibility test for CAM

	Nested PCR-QP		
	Mutation (n = 7)	Wild type (n = 14)	Not tested (n = 2)
DST for CAM			
Resistant (n = 7)	7	0	0
Sensitive (n = 16)	0	14	2

*PCR* polymerase chain reaction, *QP* Q probe, *DST* drug susceptibility test, *CAM* clarithromycin

The coincidence rate for Nested PCR-QP and drug susceptibility testing is shown in Table 3. Of the 23 adults who underwent the tests, we were able to use Nested PCR-QP to analyze 23S rRNA genetic mutations in 21 who tested positive in the stool *H. pylori* antigen test. The CAM resistance mutation results for all of these 21 adults were consistent with the results of drug susceptibility testing in culture tests.

## Discussion

Our novel Nested PCR-QP method uses the remnant solution of the stool *H. pylori* antigen test. This enables simultaneous identification of the presence of the *H. pylori* gene and CAM resistance mutations without the need to perform EGD. Therefore, this method may be a useful aid for drug selection to eradicate *H. pylori*.

Although an eradication therapy regimen is generally selected after determining whether the patient has CAM resistance using enrichment culture or sensitivity testing, these require tissue samples to be obtained endoscopically for use in culture and sensitivity testing. Many young individuals are asymptomatic, despite *H. pylori* infection [34], and highly invasive tests, such as EGD, place a major burden on the patient. Therefore, a non-invasive test that can be used in younger patients and that enables a definitive diagnosis of *H. pylori* infection using a stool antigen test reagent while simultaneously determining whether the patient is CAM resistant is highly recommended. A definitive diagnosis and determination of CAM resistance would increase the efficiency of eradication therapy. Therefore, our method is also useful in selecting a drug for eradication in that situation. Our novel method detected CAM sensitivity among *H. pylori* at a level of 93%, and the results of CAM resistance were completely consistent with drug susceptibility testing. These were good results compared with previous reports [27–29].

In our former study of third-year middle school students, the proportion of CAM resistant mutations among *H. pylori* was 52.8% (28/53), and in our latter study of adults, it was 30.4% (7/23), indicating that adolescents tended to have a higher rate of CAM resistance (*P* = 0.07). It has been reported that the CAM resistance rate of *H. pylori* is high among young people in Japan [24], which is believed to be a result of the frequent use of CAM prescriptions for childhood respiratory tract infections in this generation [35].

In subjects who underwent eradication therapy (VPZ, AMPC, and CAM) who could be followed up, we found that while the success rate for those with no CAM resistance mutation was 94.4% (17/18), the success rate for those with such a mutation was 82.6% (19/23). While there was no significant difference in the success rate between those with and without a CAM resistance mutation, there appeared to be a tendency for higher eradication therapy failure rates among those with a CAM resistance mutation, as detected by our novel method. It has been found that the success rate of *H. pylori* eradication therapy using CAM is low in CAM-resistant *H. pylori* [36], but the success rate of eradication in the CAM-resistant mutation group was 82.6% (19/23) in the present study. It is thought that this may be due to the use of VPZ, as similarly described by Murakami et al. [37]. If CAM resistance of *H. pylori* is tested by our new method before eradication treatment and the drug is changed according to the results, the eradication success rate could be higher among young Japanese patients with CAM resistance. However, there is a report recommending treatment with dual therapy using VPZ and AMPC, because a good eradication success rate can be obtained regardless of CAM resistance in *H. pylori* using VPZ [38]. In areas of high CAM resistance to *H. pylori* in Japanese adolescents, it is strongly recommended to examine CAM resistance before selecting eradication agents, and this new method seems to be very beneficial [35, 39]. In such areas, there are reports that it is recommended to use MNZ from the primary eradication therapy other than insurance medical treatment [40], but there is also a problem that MNZ resistant *H. pylori*. Although the MNZ resistance *H. pylori* rate is low in Japanese children, there are areas in the world where the MNZ resistance rate is high, so we think that it is important to accurately confirm the presence or absence of CAM resistance *H. pylori* by our novel method, and CAM should be used properly. In addition to this, the equipment required for PCR testing using our novel method is a conventional PCR equipment, and the reagents for testing cost about $25 per sample. Furthermore, the required time is 1 h for DNA extraction, 3 h for 1st PCR, 2 h for 2nd PCR, and half an hour for data analysis. Therefore, the time taken to complete a test is about 6.5 h.

This study has several limitations. The first limitation is that we were unable to investigate the effect of ingestion of gastric antacids, such as proton pump inhibitors. Second, although this new method could evaluate the CAM-resistant mutations of *H. pylori* without being affected by the susceptibility of AMPC and MNZ, we were unable to assess the effect of differences of *H. pylori* genotypes (e.g., the presence of the *cag* pathogenicity island).

## Conclusions

The novel Nested PCR-QP method is able to simultaneously identify the presence of *H. pylori* genes and the presence of CAM resistance mutations using the remnant solution from stool *H. pylori* antigen tests. In this method, EGD does not need to be performed. This method could be useful for selecting a drug aimed at reducing *H. pylori* eradication failure rates and further improving the prevention of gastric cancer.

## Data Availability

The datasets used and/or analyzed during the current study are available from the corresponding author on reasonable request.

## Abbreviations

CAM: Clarithromycin
QProbe: Quenching probe
PCR: Polymerase chain reaction
*H. pylori*: *Helicobacter pylori*
EGD: Esophagogastroduodenoscopy
VPZ: Vonoprazan
AMPC: Amoxicillin
MNZ: Metronidazole
RUT: Rapid urease test
MIC: Minimal inhibitory concentration
UMIN: University Medical Information Network
